# Validity and accuracy of a machine learning predictive model in the exploitation of patient-related outcomes in spine surgery

**DOI:** 10.3389/fsurg.2025.1710512

**Published:** 2026-01-09

**Authors:** Arthur André, Bruno Peyrou, Jean-Jacques Vignaux, Louis Boissière, Ibrahim Obeid

**Affiliations:** 1 Ramsay Santé, Clinique Geoffroy Saint-Hilaire, Paris, France; 2 Research Department, Cortexx Medical Intelligence, Paris, France; 3 Spine Clinic, Polyclinique Jean Villar, ELSAN Group, Bordeaux, France

**Keywords:** spine surgeries, artificial intelligence, machine learning, patient-reported outcome measurement (PROM), minimal clinically important differences (MCID)

## Abstract

**Background:**

Lumbar spine disorders are among the most prevalent and disabling conditions worldwide. Patient selection for surgery remains highly complex, and the benefits of surgical interventions remain uncertain, potentially depending on patients’ baseline health characteristics. Patient-related outcome measurements represent a standard method for assessing treatment success in lumbar surgery. The aim of this study is to prospectively validate the accuracy of a deep learning algorithm in predicting the clinical outcomes of patients undergoing lumbar surgery [minimal clinically important difference (MCID)/no-MCID].

**Material and methods:**

This study is multicentric, longitudinal, and prospective study was conducted over a 16-month period (September 2021 to December 2022). Patients with a surgical indication for lumbar decompression were included preoperatively and enrolled in the Surgery Medical Outcomes (SuMO©) mobile application to fill in the preoperative and postoperative data. Patients were classified into two categories according to their postoperative outcomes. The MCID was defined using the Oswestry Disability Index (ODI), combined with the intake of opioids and the presence of motor loss in patients. These results were then compared to the prediction of the algorithm based on preoperative data to determine the accuracy of the algorithm.

**Results:**

A total of 119 patients were enrolled preoperatively, and postoperative follow-up data were obtained for 103 patients. The mean preoperative ODI was 0.43 (SD 0.17). The postoperative mean ODI was 0.28 (SD 0.18) at 1 month and 0.14 (SD 0.16) at 3 months. At 8 months, the mean postoperative ODI in the MCID group was 0.12, while it was 0.26 in the no-MCID group. The algorithm predicted the outcome with an accuracy of 81.6% (receiver operating characteristic score).

**Conclusion:**

This study confirms the validity and accuracy of the algorithm in prospectively predicting postoperative outcomes, as well as the sensitivity of the MCID definition, especially when coupled with remote, patient-centered follow-up. Artificial intelligence-based algorithms may help physicians in their future daily practice by addressing personalized patient care.

## Introduction

Lumbar spine pathologies are among the most disabling disorders worldwide, and numerous factors contribute to this pathological condition (1, 2). Precision medicine aims to consider each patient's personal characteristics to select the most appropriate treatment (3).

Lumbar spinal stenosis (LSS) is caused by a gradual narrowing of the spinal canal associated with degenerative changes or disc herniation, and it has become one of the most common indications for spinal surgery (4).

The use of surgery to treat lumbar spinal stenosis has increased over recent decades, as has the precision of surgical procedures (5). These advances aim to improve surgical workflow, patient safety and efficiency, personalized patient care, and, in some cases, minimize the potential risk of future instability and deformity (6).

Patient-reported outcome measures (PROMs) are currently considered the gold standard for evaluating long-term outcomes following spine surgery (7, 8), and they also play a central role in assessing cost-effectiveness across treatment pathways (8). Among these tools, the Oswestry Disability Index (ODI) [a validated 10-item questionnaire that quantifies functional disability on a scale from 0 (no disability) to 100 (maximal disability)], is one of the most widely used PROMs, offering a validated and comprehensive assessment of the functional status of a patient in daily life. Because of its relevance and sensitivity, the ODI is often incorporated into the definition of the minimal clinically important difference (MCID), a threshold that reflects meaningful clinical improvement after surgery.

The MCID has become a key concept in both clinical evaluation and predictive modeling, particularly in the context of lumbar spine surgery. However, the optimal method for defining MCID remains a topic of debate. Thresholds vary across studies and across different PROM instruments, and no consensus has been reached regarding the most appropriate calculation method (9). Recent research has shown that the definition of MCID—whether based on ODI, COMI, or pain-related scales—can significantly impact the results of predictive models (10).

Collecting standardized PROMs, such as the ODI, with high frequency and accuracy is therefore essential for supporting the development of reliable predictive models using machine learning (ML). Such structured data streams enable advanced algorithms, including deep neural networks (DNNs), to establish associations between preoperative patient characteristics and long-term outcomes and, ultimately, to predict the achievement of MCID (11).

The clinical evolution after spine surgery is a major issue in determining the relevance of care of a patient. The ability to preoperatively predict which patients will achieve an MCID—i.e., a significant improvement after lumbar spine surgery, could improve the relevance of surgical indications and follow-up.

Artificial intelligence (AI) and predictive ML in spine surgery rely on a wide variety of models (12), including random forest (13), gradient boosting machines (GBMs) (14), or artificial neural networks (ANNs) (15, 16). Those models have been used to assess the risks of complications (17), predict patient outcomes (18–20) such as MCID (21), estimate the likelihood of readmission (22), or support surgical decision-making (23), including in certain cases helping evaluate the treatment (24, 25). A recent study compared different predictive models to predict MCID based on the Quality Outcomes Database (QOD Study) (21).

However, the benefits and applications of artificial intelligence tools in everyday clinical practice are not yet fully available to surgeons. AI could be a useful tool for exploring new clinical determinants evaluated by traditional studies (26, 27), as well as through the use of specialized generative AI, such as chatbot assistants. Only a few tools have been validated by the FDA for use in daily practice (28). Most AI/ML tools are used in radiology (29).

To improve patient care and unlock daily innovative tools, studies suggest that a greater emphasis should be placed on the distinctive characteristics of AI/ML when defining new AI/ML-based medical devices (30).

To predict patient long-term quality of life (QoL) in a day-to-day practice, we developed a platform that integrates a previously validated deep neural network algorithm (15) with a mobile application. This application prompts patients to fill medical, paramedical, and socio-professional information to establish a preoperative clinical table to complete PROMs at regular intervals to track the evolution of outcomes after surgery.

A previous study on a retrospective cohort showed an accuracy of 0.8 in predicting surgery outcome at 1 month (15).

To evaluate the validity and accuracy of our predictive model, we conducted a prospective study comparing 6-month actual outcomes with the predictions generated by our algorithm.

## Methods

Patients were enrolled between September 2021 and July 2022 at two Spine centers in Paris (Clinique Geoffroy-Saint Hilaire) and Bordeaux (Clinique Jean Villar) after a preoperative surgical clinic.

After obtaining informed consent, only patients who had not objected to the use of their anonymized health data were included in the analysis.

Inclusion criteria were as follows:

- Adult >18 years old.
- Eligible for lumbar decompression surgery, whether instrumented or not.
- Covered by social insurance.
- Provided informed consent.

Exclusion criteria were as follows:

- Patients under 18 years of age.
- Pregnant or breastfeeding woman.
- Individuals under safeguard measures or guardianship.
- Arthrodesis involving more than two levels.
- Interventions linked to a traumatic or infectious context.

### Ethics statement

This clinical study was conducted in compliance with ethical standards and was approved by the relevant Institutional Review Board(s) and Ethics Committee(s). Specifically, the study protocol received approval from the French Ethics Committee (Commission de Protection des Personnes). The study was also registered at the Drug and Medical Devices French Agency under the identifier IDRCB 2021-A00055–36 and was listed on ClinicalTrials.gov under the registration number NCT05166018.

Written informed consent was obtained from all participants prior to their inclusion in the study. Participants received detailed information regarding the study objectives, procedures, potential risks, and benefits, in accordance with the principles outlined in the Declaration of Helsinki. All data were anonymized to ensure confidentiality and privacy.

All developments carried out were compliant with the General Data Protection Regulation.

### Data collection

**Figure 1:**
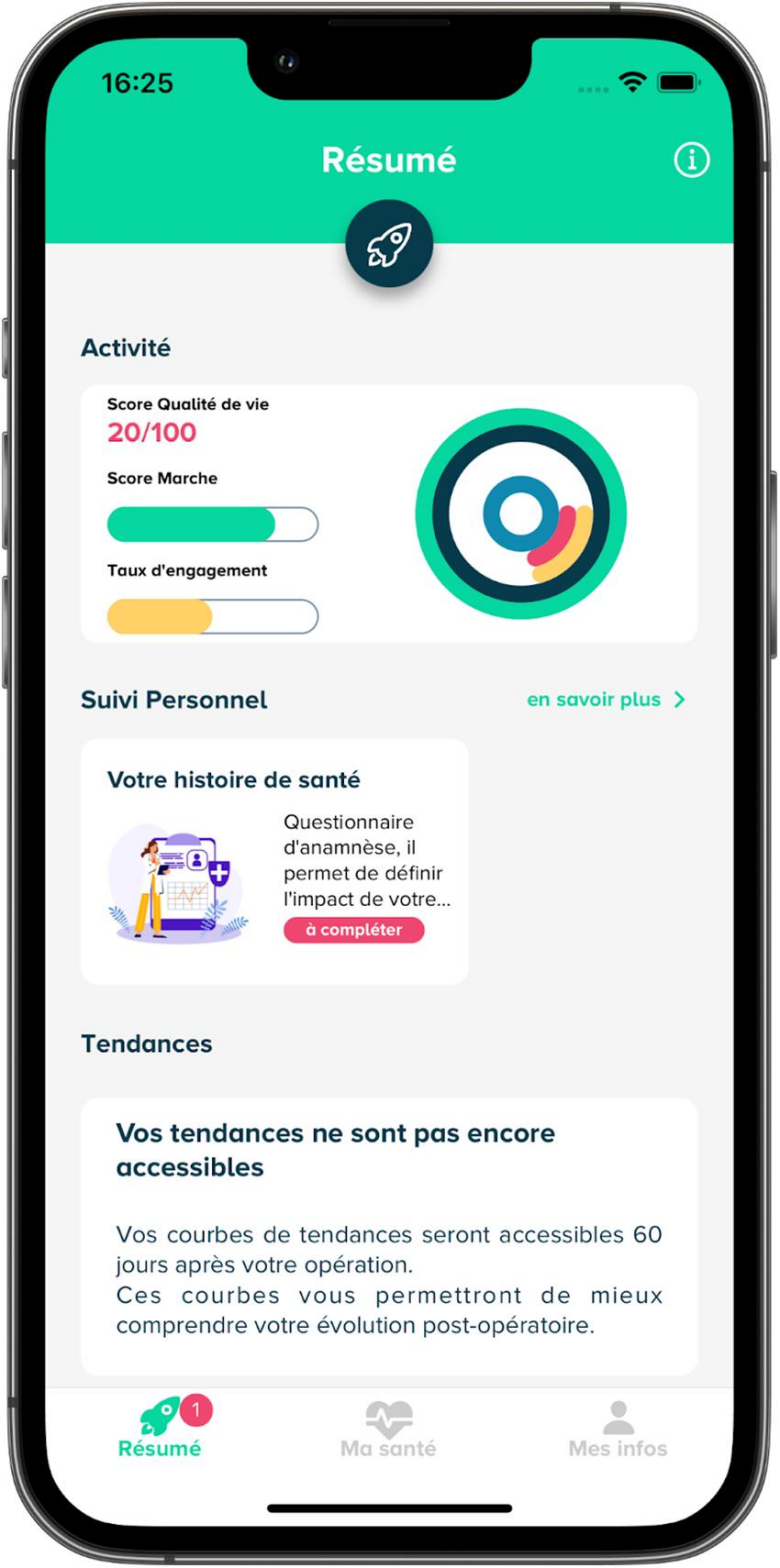
Homepage SuMO application—this screen shows the main activity of the patient daily use of the SuMO device. Patients can self-evaluate their follow-up by completing personal profile-driven questionnaires, such as PROM, and see their health trends.

**Figure 2:**
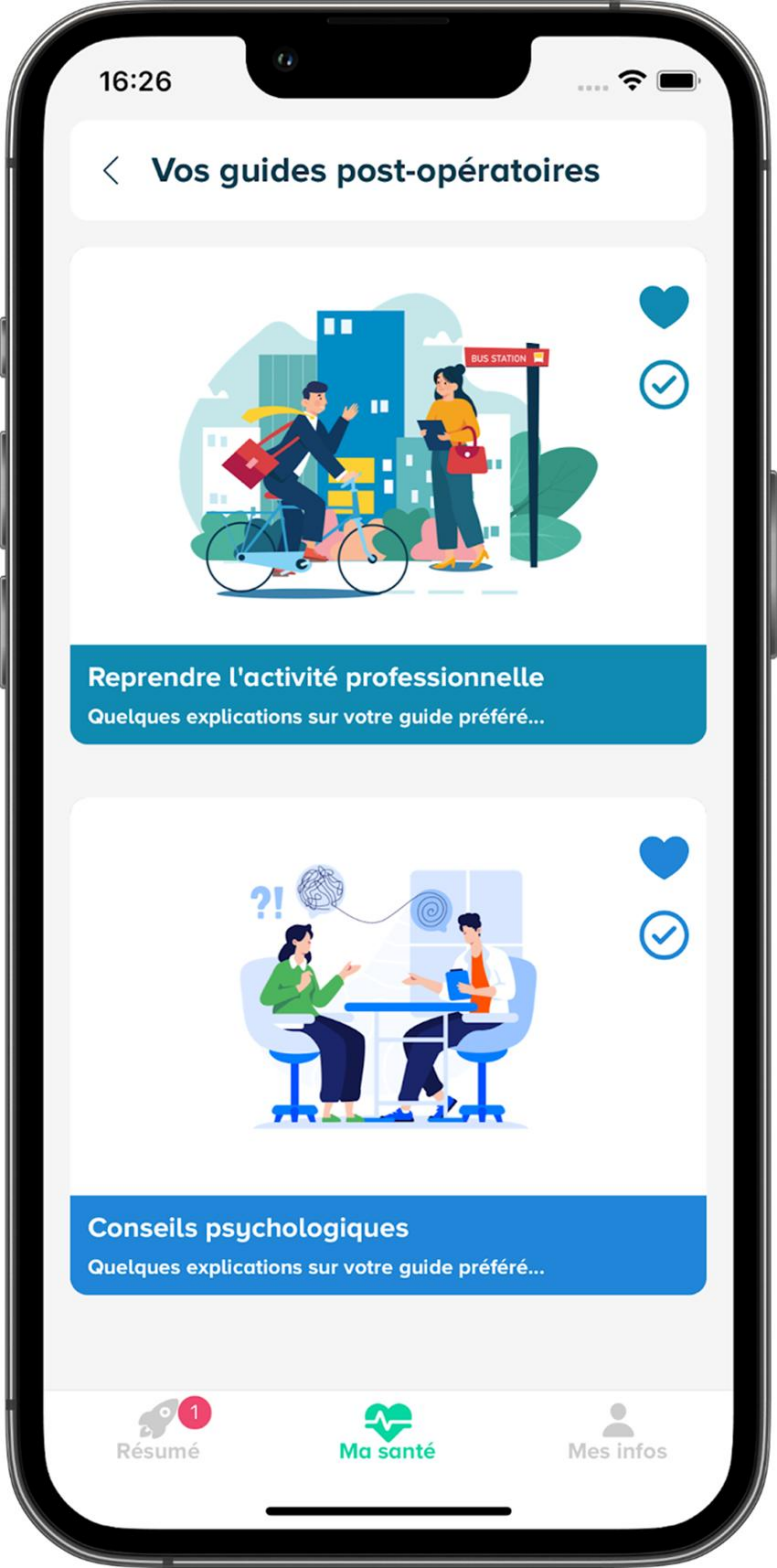
SuMO application activity—this screen shows the main action of the patient during their daily self-care. Patients can benefit from access to information and personalized advice tailored to their personalized data.

To evaluate model prediction on standardized data, we developed a dedicated data collection platform—Surgery Medical Outcomes (SuMO) and its associated patient mobile application (see Figures 1, 2).

SuMO is a CE-marked software as a medical device (SaMD) that uses machine-learning-based predictive models to support clinical decision-making throughout the spine surgery care pathway. The platform aggregates pre-, peri-, and postoperative clinical, radiological, and patient-reported data to (i) estimate individual surgical risk profiles; (ii) suggest optimal surgical strategies; (iii) monitor postoperative recovery through longitudinal PROMs, medication use, and imaging; and (iv) deliver tailored educational content and alerts to patients during follow-up to support safe recovery and improve quality of life.

In the present study, SuMO was used exclusively as a digital platform to collect PROMs and clinical follow-up data; its predictive recommendations were not used to guide or modify clinical care.

To reduce negative effects and raise patient awareness of their actions during the episode of care, data were collected via questions associated with the episode of care (see Figure 1).

**Figure 3:**
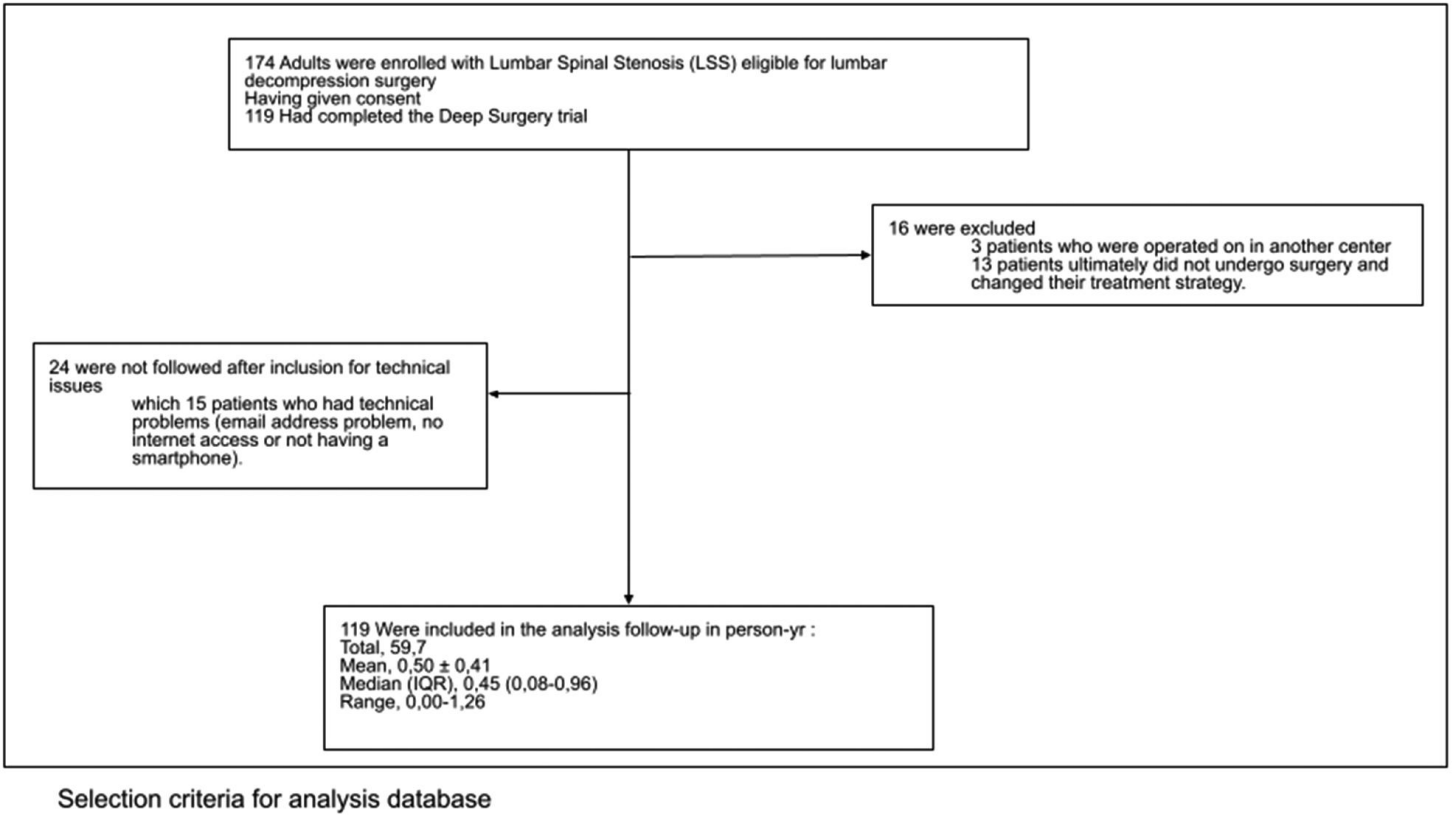
NRS leg pain follow-up—points are mean values of NRS leg pain for intervals of days [preop, (3; 15 days), ([15; 30 days) …] for all patients in blue, for the MCID group in green, and for the no-MCID group in orange. Polynomial regressions of NRS leg pain are also plotted for the three groups.

**Figure 4:**
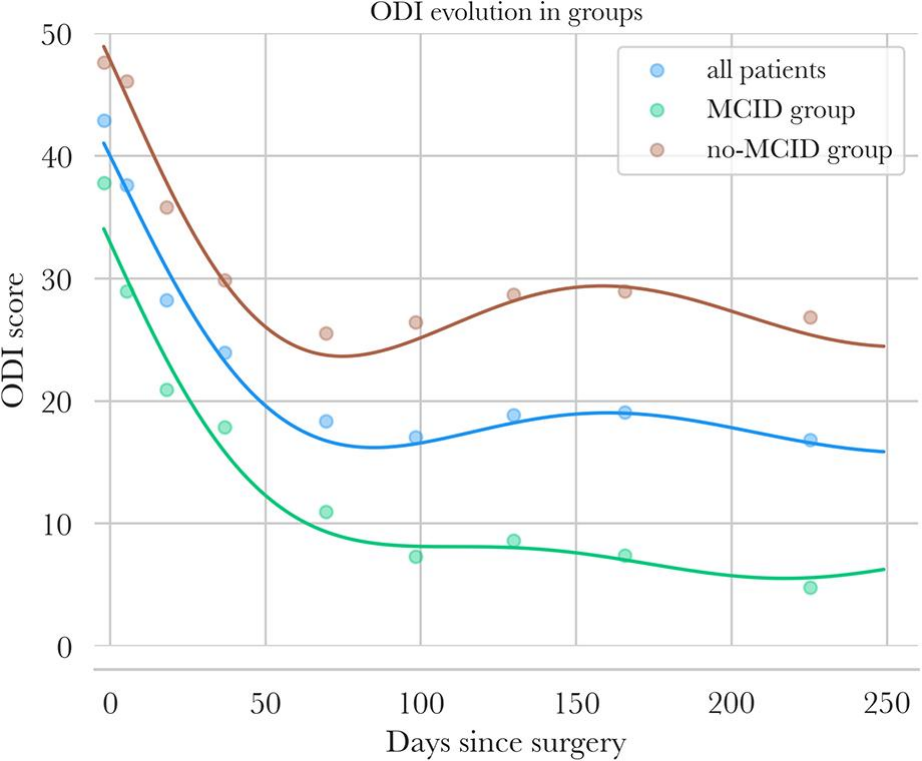
NRS low pain follow-up—points are mean values of NRS low pain for intervals of days [preop, (3; 15 days), (15; 30 days) …] for all patients in blue, for the MCID group in green, and for the no-MCID group in orange. Polynomial regressions of NRS low pain are also plotted for the three groups.

**Figure 5:**
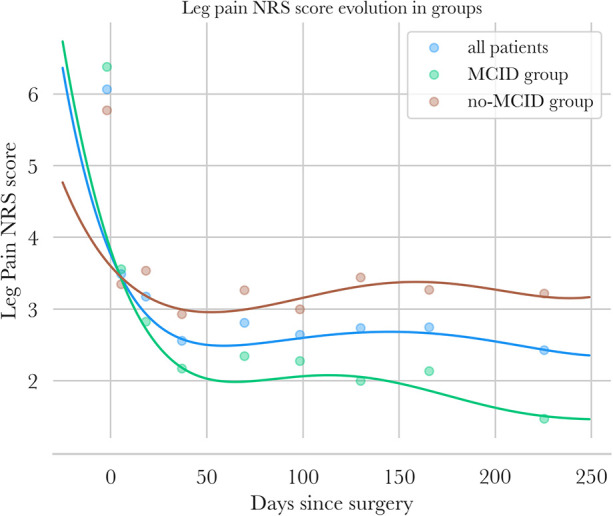
Clinical categorical outcome follow-up—points are mean values of categorical outcome declaration for intervals of days [preop, (3; 15 days), (15; 30 days) …] for all patients in blue, for the MCID group in green, and for the no-MCID group in orange. Polynomial regressions of categorical outcomes are also plotted for the three groups.

Preoperative patient characteristics [demographic characteristics, pathological characteristics, imaging features, conditions (history and comorbidities), drug treatments, socio-professional characteristics, physiological characteristics (stress, physical activity, etc.) (see Table 2)], and postoperative data such as PROMs [ODI, numeric rating scale (NRS) Pain] (see Figures 3–5) (at days +3, 15, 30, 45, 60, 75, 90, 120, 150, 180, 210, 240, 270, 300, 330, 360), as well as information on resumption of professional activity, sleep, physical activity, and feeding, were collected through this application.

Perioperative data were collected from medical reports by a clinical research associate (hospitalization, surgical procedure, and intraoperative parameters; see Table 3). In this cohort, 42% of procedures were performed for soft disc herniation with radicular conflict, while the remaining cases involved degenerative posterior facet arthropathy and disc degeneration. The choice of surgical technique (discectomy vs. decompression with or without instrumented fixation) was left to the discretion of the operating surgeon.

Usability was evaluated by the System Usability Scale (SUS) questionnaire (31, 32).

### Minimal clinically important difference

During our first study, we chose a minimal clinically important difference (MCID) based on retrospectively collected clinical outcomes to develop and test our deep learning algorithm (15), such as walking distance, motor and sensitivity recovery, anxiety–depression syndrome, and neuropathic pain.

To have a sufficiently relevant MCID in a prospective study and ensure reliable PROM collection, we chose to improve our definition of long-term MCID.

The sensitivity signs improve slowly; the time needed for this criterion to stabilize is incompatible with the goal of this study to rapidly determine the long-term outcomes of the patient.

Anxiety–depression syndrome and neuropathic pain during follow-up were collected only during the perioperative period; therefore, these variables were not retained as criteria.

In this study, surgery success or failure was defined using a composite MCID based on three criteria (see Table 1).

**Table 1:** MCID used to provide exhaustive information on the general evolution of the patient prospectively.

Variable evaluated as MCID	Value
Motor difficulty when walking or moving the leg	Binary criteria (1 no-MCID; 0 MCID)
Regular consumption of opioids or anti-inflammatory drugs	Binary criteria (1 no-MCID; 0 MCID)
ODI	<0.2 MCID/>0.2 no-MCID

If the patient presents with at least one criterion, the MCID was considered not reached.

A MCID is a threshold used to measure the effect of clinical treatments. Various threshold values have been proposed as MCID for different PROM instruments, despite a lack of real consensus on the optimal MCID calculation method (9).

If the patient presents with at least one criterion, the MCID was considered reached.

### AI predictive model

The AI predictive model used in this study was based on a deep learning architecture employing an ANN. The model was initially developed and validated on a retrospective cohort and was later trained using a large dataset of synthetic patient records to enhance training diversity and robustness. These synthetic records were generated from real electronic health records (EHRs), with simulated variations across 68 binary-encoded pre- and perioperative clinical, radiological, and psychosocial variables. Each variable was weighted according to its estimated influence on long-term surgical outcomes. The final synthetic dataset included 10,000 training cases and 2,000 test cases, with a balanced distribution of outcomes (15).

The network architecture comprises multiple hidden layers using rectified linear unit (ReLU) activation functions and a sigmoid output node to classify patients into two prognostic categories: favorable (MCID) and unfavorable (no-MCID). The training was performed using binary cross-entropy as the loss function and the Adam optimizer. Further details on model architecture and training protocol are available in our previous study.

For this prospective evaluation, the model was applied without modification to real patient data collected through the SuMO platform. We used a complete-case analysis strategy, restricting the testing dataset to patients with a follow-up period exceeding 90 days, thus allowing for a reliable comparison of outcomes. The model used both preoperative and perioperative variables to predict long-term MCID achievement, supporting its potential for integration into real-time clinical workflows.

### Statistical analysis

Data processing and statistical analysis were conducted using Python (v3.9), with Pandas (v1.5.3) and NumPy (v1.24.4) for data manipulation and SciPy (v1.10.1) for statistical testing.

Patients were divided into two groups based on outcomes: MCID and no-MCID, according to a composite criterion (ODI < 0.2, absence of motor deficit, and no regular use of opioids or anti-inflammatory medications). To evaluate differences in clinical characteristics between these two groups, variables were categorized as either continuous or dichotomous.

For continuous variables, Pearson's correlation and linear regression were used to assess intervariable associations, while group comparisons were performed using chi-squared tests of independence. For associations between categorical variables, chi-squared tests were applied. When analyzing relationships between categorical and continuous variables, point–biserial correlations were calculated.

To explore the most relevant features associated with postoperative outcomes, each variable was individually compared between the MCID and no-MCID groups using the tests outlined above. Statistical significance was set at *α* = 0.05 (two-tailed).

For postoperative longitudinal variables such as ODI, NRS for leg pain (NRS-LP), NRS for back pain (NRS-BP), and other PROMs collected at regular intervals, linear interpolation was applied to impute missing intermediate values when at least two surrounding data points were available. No extrapolation was performed beyond the final available patient input; patients who stopped entering follow-up data were excluded from further timepoint analyses.

To assess model performance, predictions from the ANN were compared with actual outcomes using standard classification metrics: sensitivity, specificity, positive predictive value (PPV), negative predictive value (NPV), and global accuracy. A receiver operating characteristic (ROC) curve was generated, and the area under the curve (AUC) was calculated to evaluate overall model discrimination. A confusion matrix was constructed to assess prediction performance across both classes.

### TRIPOD-AI

The present article follows the Transparent Reporting of a multivariable prediction model for Individual Prognosis Or Diagnosis (TRIPOD) guidelines for reporting the development and validation of the prediction model (33).

## Results

### Population

**Figure 6:**
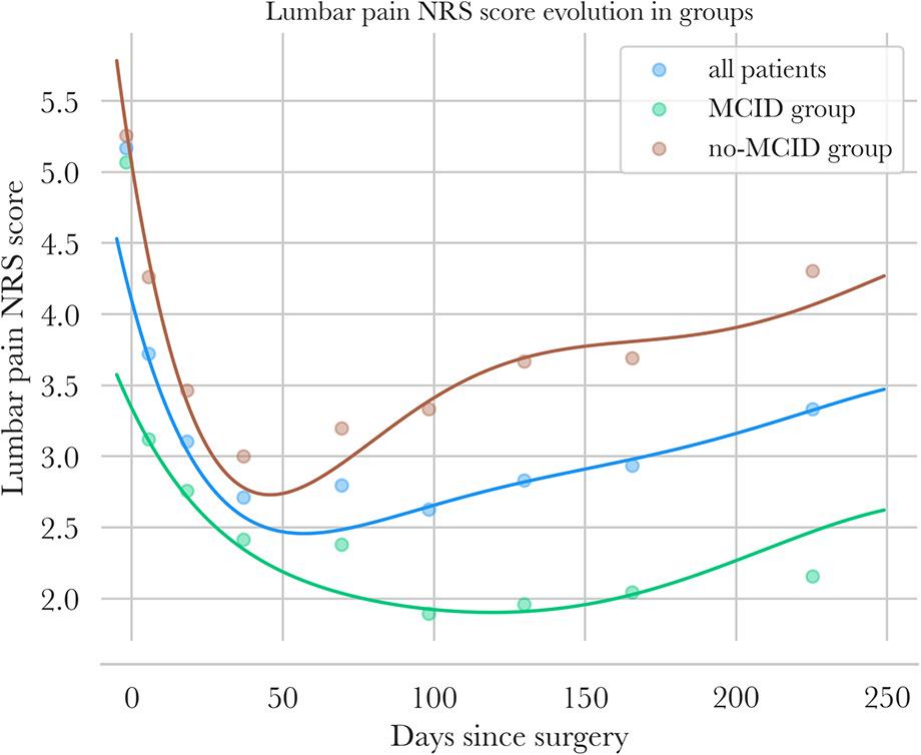
Selection criteria for analysis of databases.

A total of 174 patients were included in this study. Three patients who were operated on in another center were excluded. An additional 24 patients were lost to follow-up, and 15 patients experienced technical problems (email address problem, no internet access, or not having a smartphone). Thirteen patients ultimately did not undergo surgery and changed their treatment strategy (see Figure 6).

A total of 119 patients were followed to the end of the study with complete EHRs. Table 2 presents the baseline preoperative characteristics of patients, and Table 3 presents the perioperative characteristics of patients recorded during back surgery. For each variable, the mean, median, standard deviation, and number of respondents are reported.

**Table 2:** Baseline preoperative characteristics of patients undergoing back surgery before surgery.

Variable	Mean ± SD (*N* total)	Median
Demographic characteristics		
Age (years)	49.34 ± 14.08 (119)	48
Sex, % (no.)		
Male	61 (73)	
Female	39 (46)	
Body mass index (BMI), mean	26.74 ± 4.7 (119)	25,57
Socio-professional, % (no.)		
Sedentary work	32 (119)	
Heavy activity at work	45 (119)	
Moderate activity at work	53 (119)	
Nomad activity at work	47 (119)	
Number of days off work before surgery (days)	45.78 ± 81.46 (119)	7
Work stress	44.14 (111)	
Family problematics	28.57 (112)	
Physiological characteristics, % (no.)		
Sleep disorder(s)	42.02 (119)	
Appetite disorder(s)	10.17 (118)	
Practicing a specific sport	27.97 (118)	
Feeling depressed	19.27 (109)	
Metabolic equivalent of task		
<4	12 (112)	
4–7	40 (112)	
7–10	41 (112)	
10	7 (112)	
Pathological characteristics, % (no.)		
Days with pain before surgery (days)	45.78 ± 81.46 (119)	7
Acute, % (no.)	80.4 (78)	
Chronic, % (no.)	19.6 (19)	
ODI	41 ± 17 (111)	38
NRS-LP (0–10)	5.46 ± 2.88 (111)	6
NRS-BP (0–10)	5.04 ± 2.23 (111)	5
Bilateral sciatica	13.45	
Walking perimeter reduction	64.66 (116)	
Feeling of motor loss associated with the episode	39.13 (115)	
Feeling of sensitive loss associated with the episode	32.17 (115)	
Impulsive movement associated with the episode	18.18 (110)	
Onset during the night	69.57 (115)	
Back stiffness associated with the episode	48.11 (106)	
Sphincteric troubles associated with the episode	8.55 (117)	
Pathological characteristics—imagery features, % (no.)		
MODIC from the imaging report	43.37 (83)	
Disk calcification from the imaging report	12.94 (85)	
Stenosis from the imaging report	19.05 (84)	
Protrusion from the imaging report	42.11 (76)	
Excluded hernia from the imaging report	13.7 (73)	
Non-excluded hernia from the imaging report	70.8 (113)	
Arthritis from the imaging report	43.52 (108)	
Articular hypertrophy from the imaging report	5.06 (79)	
Osteophytis from the imaging report	3.85 (78)	
Spondylolisthesis from the imaging report	3.57 (84)	
Lumbar level operated		
L1L2	1 (98)	
L2L3	4 (98)	
L3L4	5 (98)	
L4L5	55 (98)	
L5S1	35 (98)	
Other condition features, % (no.)		
Historic previous spine surgery	20.17 (119)	
Smoking	15.25 (118)	
Historic opioid consumption	29.57 (115)	
Chronic digestive troubles	14.91 (114)	
Diabetes	5.04 (119)	
Depression	10.17 (118)	
Sleep apnea	4.24 (118)	
Chronic obstructive pulmonary disease (COPD)	1.69 (118)	
Liver disease(s)	0 (119)	
Atherome(s)	5.08 (118)	
Kidney disease(s)	0 (118)	

**Table 3:** Baseline perioperative characteristics of patients—these data are obtained by the exploitation of medical reports by clinical research associates.

Variable	Mean ± SD (*N* total)	Median
Stay characteristics		
Number of days of hospitalization before surgery (days)	0.8 ± 0.4 (119)	1
Length of stay (LOS) (days)	2.15 ± 2.32 (118)	2
Positioning in the day's calendar	2.24 ± 1.27 (90)	2
Length of surgery procedure (min)	68.09 ± 33.51 (119)	75
Physiological hospitalization characteristics, % (no.)		
Alimentation back to normal	94.68 (94)	
Sleep back to normal	94.62 (93)	
Bleeding during hospitalization	26.05 (119)	
Infection episode during hospitalization	0 (118)	
Postoperative complications during hospitalization	2.54 (118)	
ASA score	1.69 ± 0.66 (108)	2
NYHA score	1.07 ± 0.25 (30)	1
Metabolic equivalent of task <4	0.06 (83)	
Metabolic equivalent of task between 4 and 7	0.58 (83)	
Metabolic equivalent of task between 7 and 10	0.34 (83)	
Metabolic equivalent of task = 10	0.02 (83)	
Pathological characteristics, % (no.)		
Back to normal autonomous walk	94.78 (115)	
Anti-inflammatory consumption	68.38 (117)	
Opioid consumption after surgery	27.59 (116)	
Neuropathic pain after surgery	12.82 (117)	
Other conditions during hospitalization, % (no.)		
Anxiety episode after surgery	1.74 (115)	
Rehabilitation center after hospitalization	0.87 (115)	

ASA, American Society of Anesthesiologists; NYHA, New York Heart Association.

Of the 119 patients included, 61 had 90 days of follow-up and provided data. Among these, 29 (47%) patients were good recipients of surgery (MCID, as defined in Table 1) and 32 (53%) patients were not (no-MCID).

### Follow-up

#### ODI

**Figure 7:**
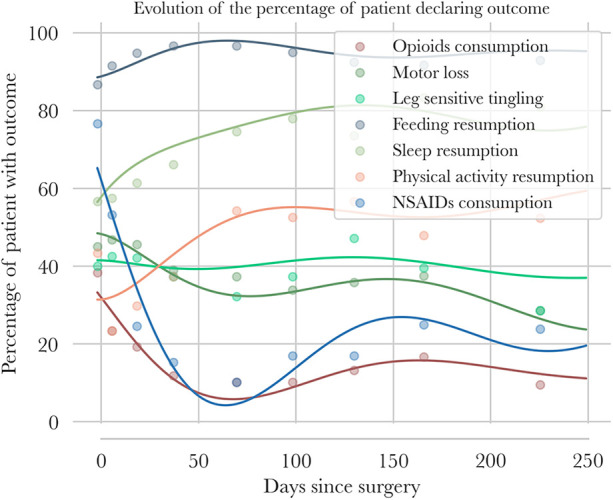
Oswestry Disability Index follow-up—points are mean values of ODI for intervals of days [preop, (3; 15 days), (15; 30 days) …] for all patients in blue, for the MCID group in green, and for the no-MCID group in orange. Polynomial regression of ODIs is also plotted for the three groups.

ODI scores of patients are presented in Figure 7.

In the overall population, ODI showed a continuous decrease from 43 to 17 at 90 days (−60%) and then stabilized at 8 months after surgery, showing the global positive impact of the surgical procedure on the long-term QoL.

ODI scores decreased continuously from baseline to 90 days after surgery, falling from 48 to 26 in the no-MCID (−46%) group and from 38 to approximately 7 in the MCID group (−82%). Then, the two groups behave differently: in the no-MCID group, ODI stabilized at approximately 27 by 240 days (−44% overall), whereas in the MCID group, ODI continued to decrease, reaching approximately 5 (−87% overall).

#### Pain

NRS pain scores of patients are presented in Figures 3, 4.

Leg pain decreased in both groups until 2 months, reaching 2.3 in the MCID group (−64% from baseline) and 3.3 in the no-MCID group (−43%). Beyond that point, pain scores stabilized and slowly decreased until 8 months, reaching approximately 1.5 in the MCID group (−76% overall) and 3.2 in the no-MCID group (−45%) (see Figure 10 in the Appendix).

Low back pain decreased in both groups until the 3-month mark, reaching 1.9 in the MCID group (−63% from baseline) and 3.3 in the no-MCID group (−38%). After 3 months, average low back pain began to rise again, reaching 2.2 at 8 months in the MCID (−57% overall) group and 4.3 in the no-MCID group (−19% overall). The overall trends were similar in both the MCID and no-MCID groups; however, the rebound was weaker in the MCID group. Ultimately, the final improvement was −57% for the MCID and only −19% for the no-MCID population.

### Clinical outcomes

Categorical outcomes after surgery are presented in Figure 5.

Nonsteroidal anti-inflammatory drugs (NSAIDs) and opioid intake decreased quickly to under 10% at 3 months and remained globally stable up to 8 months. Motor loss and leg tingling were relatively stable and oscillated around 40%, with a tendency to decrease toward 30% by 8 months.

Feeding returned to near 100% after 1 month, while sleep quality improved rapidly and stabilized at approximately 80% after 2–8 months. Return to physical activity was achieved by 60% of the population after 3 months.

#### Prediction of the model

**Figure 8:**
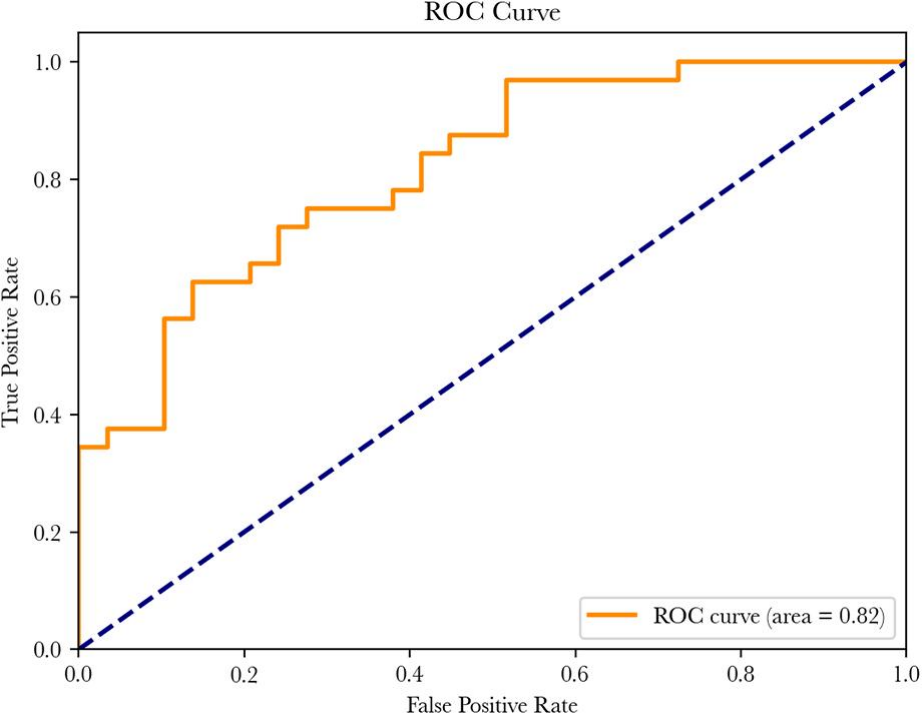
AUC of the model on prospective data.

The model showed an accuracy of 70%, with an ROC score of 0.816. Sensitivity was 81%, specificity was 59%, PPV was 68%, and NPV was 74% (see Figure 8 and Table 4).

**Table 4:** Confusion matrix of the model predictions on the cohort.

Variable	Precision	Recall	F1 score	Support
No-MCID (see Table 2)	0.68	0.81	0.74	32
MCID (see Table 2)	0.74	0.59	0.63	29
Accuracy			0.70	61
Macro-avg	0.71	0.70	0.70	61
Weighted avg	0.71	0.70	0.70	61
Sensitivity	0.81			
Specificity	0.59			
PPV	0.68			
NPV	0.74			
ROC AUC score	**0**.**816**			

Bold value indicate the main result of machine learning tools.

### Risk factors and characteristics for MCID or no-MCID outcomes

In Table 5, the criteria are displayed for the two groups of patients (MCID/no-MCID). Variables are sorted in ascending order of *p*-values to highlight the most prominent criteria for the long-term prognosis (MCID/no-MCID) of the patients. These criteria were practicing a specific sport, stenosis from imaging data, motor loss during the episode, arthritis from imaging data, stress and family conflict, metabolic equivalent of task (MET) of 10, MET between 7 and 10, metabolic equivalent of task between 4 and 7, metabolic equivalent of task <4, appetite disorder(s), and history of previous surgery (*p* < 0.05) (see Table 5).

**Table 5:** Characteristics of MCID and no-MCID participants on continuous (*t*) and categorical predictors (*χ*^2^).

Continuous variables	Mean MCID (*N*)	Mean no-MCID (*N*)	*t*	*p*	df
Number of days with pain before surgery (days)	37.04 (28)	45.2 (32)	0.29	0.13	29
ODI score	38 (29)	48 (31)	−0.23	0.23	29
Age (years)	49.9 (29)	47.41 (32)	−0.18	0.35	29
NRS-LP (0–10)	6.38 (29)	5.77 (31)	−0.09	0.64	29
Number of days off work before surgery (days)	26.66 (29)	70.09 (32)	0.05	0.79	29
Body mass index (number of points)	25.47 (29)	28.8 (32)	0.03	0.86	29
NRS-BP (0–10)	5.07 (29)	5.26 (31)	−0.02	0.92	29
Categorical variables	Mean MCID % (*N*)	Mean no-MCID % (*N*)	*χ* ^2^	*p*	df
Practicing a specific sport	46.43 (28)	18.75 (32)	16.09	0	2
Stenosis from the imaging report	37.5 (16)	12 (25)	15.39	0	2
Motor loss associated with the episode	32.14 (28)	58.06 (31)	8.55	0	2
Arthrosis from the imaging report	65.38 (26)	39.29 (28)	8	0	2
Stress—family conflict	23.08 (26)	44.83 (29)	5.55	0.02	2
Metabolic equivalent of task = 10	6.90 (29)	0 (32)	8.76	0.03	4
Metabolic equivalent of task between 7 and 10	48.28 (29)	31.25 (32)	8.76	0.03	4
Metabolic equivalent of task between 4 and 7	34.48 (29)	46.88 (32)	8.76	0.03	4
Metabolic equivalent of task <4	10.35 (29)	21.88 (32)	8.76	0.03	4
Appetite disorder(s)	3.57 (28)	18.75 (32)	4.84	0.03	2
Historic previous surgery	17.24 (29)	34.38 (32)	4.16	0.04	2
Disk calcification from the imaging report	21.05 (19)	9.09 (22)	3.81	0.05	2
Chronic digestive troubles	11.11 (27)	26.67 (30)	3.71	0.05	2
Excluded hernia from the imaging report	0 (15)	17.65 (17)	3.64	0.06	2
Sphincteric troubles	3.57 (28)	15.62 (32)	3.53	0.06	2
Protrusion from the imaging report	70.59 (17)	50 (20)	3.39	0.07	2
Sensitive loss	32.14 (28)	48.39 (31)	3.28	0.07	2
Sedentary work	48 (29)	34 (29)	2.44	0.12	2
Spondylolisthesis from the imaging report	0 (22)	8.7 (23)	2.19	0.14	2
Back stiffness associated with the episode	68 (25)	55.56 (27)	1.69	0.19	2
Impulsive movement associated with the episode	29.17 (24)	20 (30)	1.58	0.21	2
Sleep apnea condition	6.9 (29)	3.23 (31)	1.34	0.25	2
Atherome	3.45 (29)	9.38 (32)	1.32	0.25	2
Diabetes	3.45 (29)	9.38 (32)	1.32	0.25	2
Moderate activity at work	59 (29)	48 (27)	1.19	0.28	2
Chronic obstructive pulmonary disease condition	0 (29)	3.23 (31)	1.03	0.31	2
Lumbar level L5S1	35.72 (28)	36.67 (30)	3.72	0.44	5
Lumbar level L2L3	0 (28)	6.67 (30)	3.72	0.44	5
Lumbar level L3L4	7.14 (28)	6.67 (30)	3.72	0.44	5
Lumbar level L1L2	0 (28)	3.33 (30)	3.72	0.44	5
Lumbar level L4L5	57.14 (28)	46.67 (30)	3.72	0.44	5
Work stress	50 (28)	57.14 (28)	0.58	0.45	2
Heavy activity at work	41 (29)	48 (29)	0.55	0.46	2
Historic opioid consumption	37.04 (27)	43.33 (30)	0.48	0.49	2
Nomad activity at work	48 (29)	54 (28)	0.32	0.57	2
Articular hypertrophy from the imaging report	5.56 (18)	8.33 (24)	0.24	0.62	2
MODIC from the imaging report	63.16 (19)	58.33 (24)	0.23	0.63	2
Sleep disorder(s)	44.83 (29)	40.62 (32)	0.23	0.63	2
Walk perimeter	75 (28)	78.12 (32)	0.18	0.67	2
Depression	15.38 (26)	17.86 (28)	0.12	0.73	2
Bilateral sciatica	13.79 (29)	12.5 (32)	0.05	0.82	2
Smoking	13.79 (29)	12.5 (32)	0.05	0.82	2
Non-excluded hernia from the imaging report	85.71 (28)	87.1 (31)	0.05	0.82	2
Onset of the pain during the night	75.86 (29)	77.42 (31)	0.04	0.84	2
Osteophytis from the imaging report	5.26 (19)	4.55 (22)	0.03	0.87	2
Depression condition	10.34 (29)	9.68 (31)	0.02	0.9	2
Liver disease condition	0 (29)	0 (32)			2
Kidney disease condition	0 (29)	0 (31)			2

#### Satisfaction of platform users

The SUS questionnaire analysis shows a general satisfaction rating of 68.5 among users.

With 60 total hours spent on the questionnaires by all patients, each patient spent an average of 30 min on the questionnaires to fill in the equivalent of 178 health data points per patient during the 6-month follow-up.

## Discussion

The population analyzed in this prospective study is consistent with that observed in recent retrospective analyses of lumbar spine surgery outcomes, both in baseline characteristics and postoperative trajectories (34–36). The 6-month clinical outcomes we report align with trends described in the literature for similar interventions and follow-up durations (37–39).

In our cohort, a marked divergence was observed between the MCID and no-MCID groups, reflecting a real dichotomy in postoperative recovery, particularly in the return to patient autonomy—a finding consistent with previously published large-scale cohorts (40).

**Figure 9:**
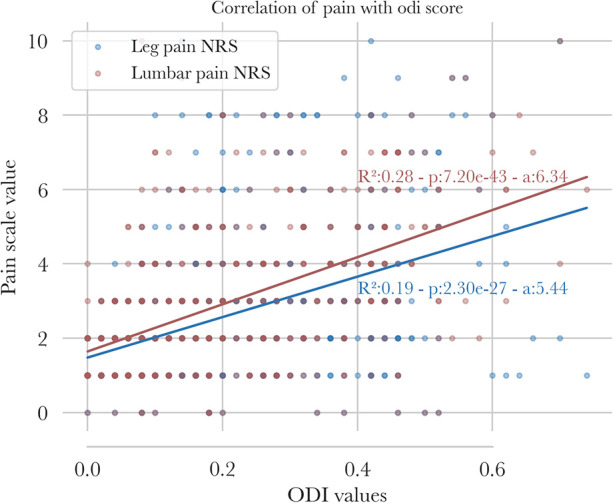
Correlation between pain intensity and disability. Scatter plot showing the relationship between ODI and NRS pain scores (leg pain and low back pain). Linear regression trendlines with associated statistics (R^2^, *p*-value, and intercept) are displayed.

**Figure 10:**
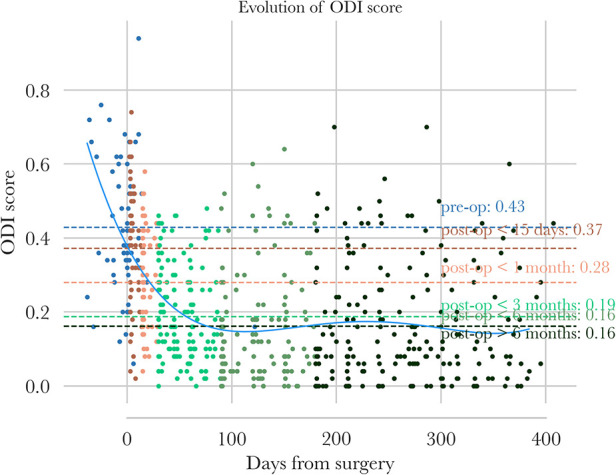
Longitudinal evolution of ODI after surgery. Scatter plot of ODI values over time (up to 400 days postoperatively). A fitted curve illustrates the overall trend, and dashed lines indicate mean ODI values across predefined postoperative time windows (preoperative, < 15 days, <1 month, <3 months, and >5 months).

Among the outcome measures, leg pain (NRS-LP) demonstrated a stronger correlation with improvements in quality of life than low back pain (NRS-BP) (see Figure 9 in the Appendix). This result aligns with prior assumptions regarding the prognostic value of radicular symptom resolution (41, 42). Although the pain delta (the absolute change in NRS score) can also serve as an indicator, its subjectivity and variability across individuals limit its reliability as a universal MCID criterion. By contrast, the ODI, which integrates functional impairment into day-to-day activities, provides a more stable and less biased measure of true postoperative recovery.

The correlation between analgesic intake and pain levels further reinforces the validity of our MCID definition. In the MCID group, pain reduction was accompanied by decreased or discontinued opioid and NSAID use, whereas persistent pain and continued drug intake in the no-MCID group reflected a more degraded quality of life. These distinctions support the use of a composite MCID definition—combining functional, symptomatic, and treatment-related parameters—to better stratify long-term surgical success.

Importantly, this definition also allowed clear segregation of postoperative categorical outcomes. Variables such as resumed physical activity, sleep recovery, persistent neurological symptoms, and analgesic consumption exhibited different behaviors in the MCID vs. no-MCID groups. These findings highlight that relying on a single dimension (e.g., pain score alone) may be insufficient and that multicriteria outcome models are essential for capturing the complex nature of recovery after spine surgery.

We also identified several individual preoperative characteristics significantly associated with favorable outcomes. Higher physical activity (as measured by the MET score), engagement in specific sports, and the absence of stress-related symptoms (e.g., family conflicts, appetite or digestive disorders) were predictive of MCID achievement. Conversely, patients with prior spine surgery or chronic comorbidities were more likely to fall into the no-MCID group. Notably, work-related variables did not show a significant predictive value in our cohort, despite their prominence in some published models.

The predictive performance of our ANN model is consistent with prior efforts to classify patients into MCID and no-MCID groups (10). However, the definition of MCID remains an ongoing point of discussion. The selection of thresholds, outcome scales (e.g., ODI vs. COMI vs. NRS), and timepoints can significantly influence predictive capacity (43, 44). Our model was validated at 6 months, a commonly accepted intermediate endpoint used in spine surgery, but longer-term follow-up (12–24 months) will be necessary to confirm the durability of both outcomes and predictions.

One of the strengths of our approach lies in the high-frequency, standardized collection of PROMs through a dedicated mobile platform. This enabled consistent data acquisition across patients and timepoints—an advantage not commonly found in previous studies relying on traditional registry-based designs. The high level of patient engagement, demonstrated by good response rates and usability scores, further supports the feasibility of embedding digital PROMs into real-world clinical pathways.

Nevertheless, several limitations must be acknowledged. First, the model was trained partly on synthetic data derived from retrospective EHRs. While this approach allowed for increased data volume and improved balance, synthetic data may not fully capture the variability of real clinical scenarios. Retraining the model on larger, fully prospective datasets would improve its generalizability and robustness. Second, although our model showed strong performance at 6 months (AUC 0.82), this time frame may be too short to capture delayed outcomes such as relapse, reoperation, or long-term functional plateau. Ongoing follow-up will allow us to assess its predictive value beyond early postoperative recovery.

Third, the sample size of our cohort remains limited for generalization. Although our results are comparable to those reported in smaller validated studies (AUC ∼0.83) (41), large-scale applications—such as those tested on broader registries—often show reduced accuracy (AUC ∼0.63) due to heterogeneous and incomplete data (10). Our approach, relying on harmonized and curated PROMs, may help mitigate these limitations, but external validation remains essential.

Alternative modeling approaches, such as logistic regression, could be explored to compare performance against ANN models, particularly given our cohort size and feature volume. However, our objective was not only to validate performance but also to build a scalable and evolutive model. Future iterations will integrate additional layers of data including imaging, genomic, and biological biomarkers (45), thus enhancing model personalization and long-term predictive strength.

The broader goal of our platform is to support a more individualized surgical decision-making process. By combining structured data collection with predictive modeling, clinicians can better anticipate outcomes, refine indications, and tailor postoperative monitoring. This anticipatory strategy may help reduce therapeutic failures—defined here as no-MCID outcomes—which are closely associated with complications, extended recovery, and patient dissatisfaction.

Such efforts align with initiatives like the International Spine Study Group (ISSG), which have shown that predictive models improve risk stratification and reduce uncertainty in surgical planning (46). AI-driven tools can complement surgeon judgment by identifying latent patterns in complex datasets, thereby enhancing both precision and confidence in care decisions (47).

## Conclusion

We found that our model demonstrated good precision in classifying long-term MCID outcomes among patients undergoing low back surgery. We also showed that an MCID definition incorporating multiple clinical outcomes is crucial for classifying patients in groups that best fit their evolution not only in terms of pain or autonomy but also regarding other outcomes like resumption of sleep, physical activity, or intake of medication. Thus, data collection via a smartphone application with high frequency represents an efficient approach for determining the long-term quality of life of patients.

The artificial neural network model used in this clinical study lends itself undeniably to holistic and multimodal prediction of long-term patient outcomes. Nonetheless, we also showed that ANN lacks an explanation for the criteria leading to the classification of patients.

The model we developed can help patients in understanding their prognosis and in tailoring a pre- and postoperative programs aimed at improving long-term quality of life. It is an essential and indispensable building block in the anticipated construction of a coherent health pathway, in terms of timing and methods, raising the standard of care with the help of artificial intelligence. A new prospective study is necessary to test the medical service to patients and enlighten the benefits of such a solution in the health pathway of patients undergoing spine surgery.

In conclusion, this study validates the real-world predictive capacity of an ANN model integrated into a mobile PROMs platform to anticipate the achievement of MCID after lumbar spine surgery. While further prospective validation and model refinement are necessary, this work illustrates a practical approach to integrating AI into daily surgical practice, promoting more personalized, data-driven, and adaptive spine care.

## Data Availability

The original contributions presented in the study are included in the article/Supplementary Material; further inquiries can be directed to the corresponding author.

## Appendix

In this Appendix, Figure 9 shows the correlation between pain intensity (NRS for leg pain and low back pain) and disability (ODI). Figure 10 shows the longitudinal evolution of the ODI score up to 400 days after surgery.
